# Fluoroscopy-free mini-PCNL using CT–ultrasound fusion guidance in a pediatric patient with recurrent cystine stones: A case report

**DOI:** 10.1016/j.eucr.2026.103367

**Published:** 2026-02-09

**Authors:** Surawach Piyawannarat, Kun Sirisopana, Yada Phengsalae, Premsant Sangkum, Wisoot Kongchareonsombat, Chinnakhet Ketsuwan

**Affiliations:** Division of Urology, Department of Surgery, Faculty of Medicine Ramathibodi Hospital, Mahidol University, Bangkok, Thailand

**Keywords:** Pediatric urolithiasis, Cystine stone, Mini-PCNL, Fluoroscopy free, CT–ultrasound fusion, Radiation free

## Abstract

Pediatric patients are particularly susceptible to ionizing radiation risks. A fluoroscopy-free mini-percutaneous nephrolithotomy (mini-PCNL) was performed using computed tomography (CT)–ultrasound fusion guidance for recurrent cystine nephrolithiasis in a 12-year-old boy. Renal access and complete stone clearance were achieved. Integrating preoperative CT with real-time ultrasonography enabled precise targeting, overcoming limitations of ultrasound-only guidance. The procedure was completed safely, with minimal blood loss and 90-min operative time. CT–ultrasound fusion-guided mini-PCNL is a feasible radiation-sparing approach for pediatric patients with complex renal stones.

## 1 Introduction

Cystinuria is a rare inherited disorder characterized by impaired renal tubular reabsorption of cystine, resulting in recurrent stone formation from childhood.^1^ Pediatric patients with cystinuria often require repeated surgical interventions, which leads to cumulative exposure to ionizing radiation. Given the increased radiosensitivity and longer life expectancy of pediatric patients and that pediatric patients with cystinuria have a high risk of lifelong stone recurrence and repeated exposure to diagnostic and therapeutic radiation,^2^^,^^3^ it is essential to apply the “as low as reasonably achievable” (ALARA) principle when planning treatment for patients in this group to minimize radiation exposure.^4^^,^^5^

Mini-percutaneous nephrolithotomy (mini-PCNL) is a popular surgical option for treating stones due to its lower incidence of serious complications and reduced need for blood transfusion compared to standard PCNL 6, 7, 8, 9. It is an established treatment for pediatric renal stones; however, the conventional fluoroscopic guidance method utilized during this procedure is a major source of intraoperative radiation.^10^ Ultrasound-guided PCNL is a widely accepted radiation-free alternative, although achieving accurate calyceal puncture can be challenging using this methodology, particularly in cases with complex renal anatomy and stone disease.^11^^,^^12^

Computed tomography (CT)–ultrasound fusion imaging, which integrates preoperative CT images showing anatomical features with real-time ultrasound guidance, may enhance puncture accuracy while avoiding intraoperative fluoroscopy. We report the use of fluoroscopy-free mini-PCNL with CT–ultrasound fusion imaging guidance to manage recurrent cystine nephrolithiasis in a pediatric patient.

## 2 Case report

**Fig. 1:**
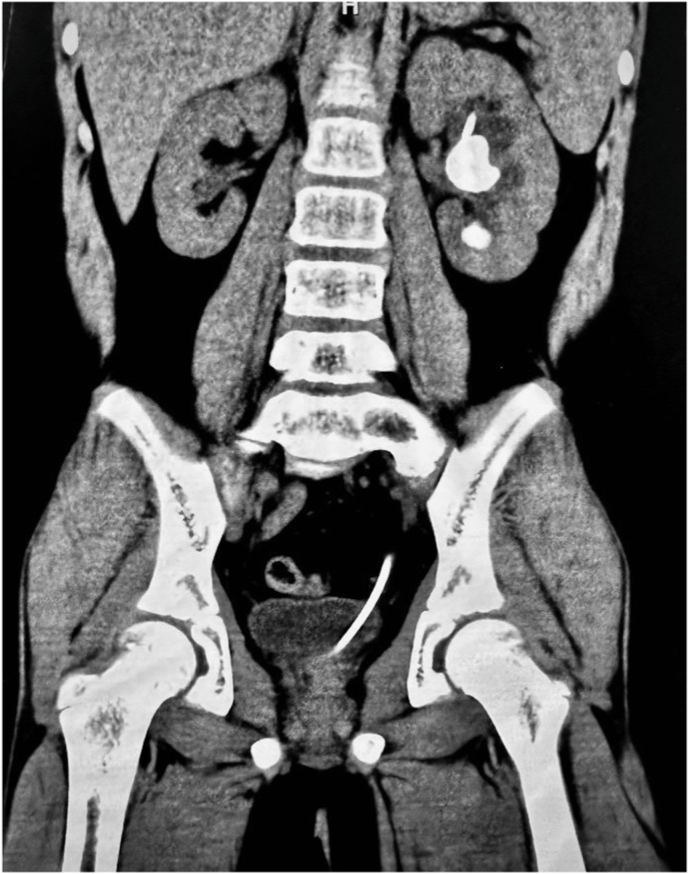
Preoperative non-contrast computed tomography image showing a 3.0 cm renal pelvic stone and a 0.7 cm lower pole stone in the left kidney.

A 12-year-old male with a known history of cystinuria presented with left flank pain. The patient's condition was previously managed with lifestyle modifications, oral alkalinization, and a right-side PCNL two years prior. A non-contrast CT scan revealed a 3.0 cm renal pelvic stone and a 0.7 cm lower pole stone in the left kidney (mean density: 720 HU; Fig. 1). A recurrent 3.5 cm staghorn calculus was also noted in the right kidney. Initial management involved inserting a left ureteral stent for pain relief before referral to our institution. Given the patient's pediatric status and stone burden, a fluoroscopy-free mini-PCNL was planned to minimize radiation exposure.

Under general anesthesia, intravenous fosfomycin was administered for perioperative prophylaxis. The ureteral stent was removed, and a 5-Fr retrograde ureteral catheter was inserted to allow saline instillation, if required.

**Fig. 2:**
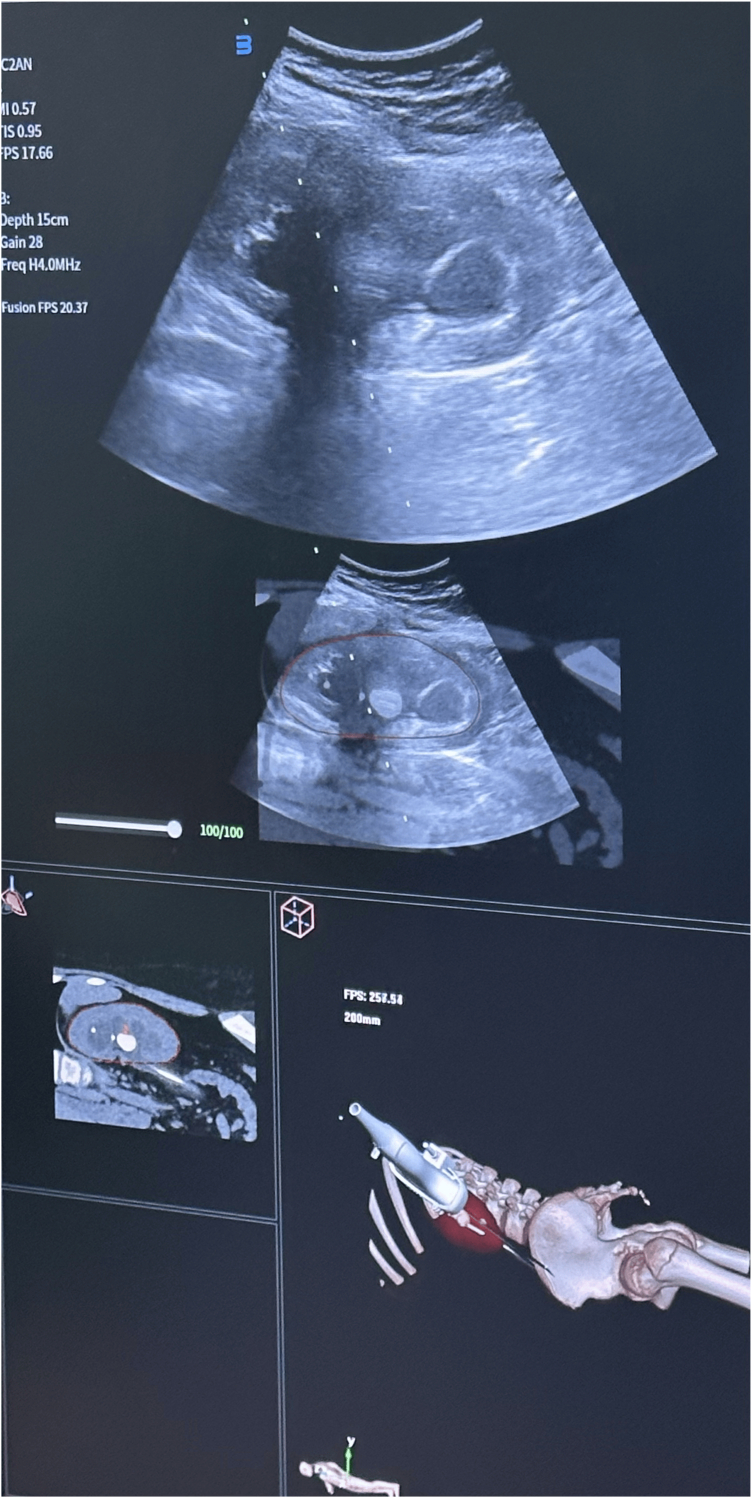
**I**ntraoperative view of the computed tomography–ultrasound fusion technique.

The patient was placed in the prone position. CT–ultrasound fusion imaging was performed using the VENUS system (Carbon [Shenzhen] Medical Device Co., Ltd., China). Preoperative CT images were imported and co-registered with real-time ultrasound images using renal anatomical landmarks. The fusion imaging enabled the precise identification of the targeted upper-pole calyx (Fig. 2).

Fusion imaging was used to guide renal access, which was achieved using an 18-gauge needle. Successful puncture was confirmed by urine efflux. A Sensor™ guidewire (Boston Scientific, Natick, MA, USA) was advanced into the collecting system under ultrasound visualization. The tract was sequentially dilated, and a 14/16-Fr suction access sheath (Clear Petra, Well Lead Medical Co., China) was inserted. A 12-Fr miniature nephroscope (MIP M system; Karl Storz, Tuttlingen, Germany) was used.

Lithotripsy was performed using a 60-W thulium fiber laser (SOLTIVE™, Olympus, MA, USA) with energy settings of 0.2–1 J and frequencies of 10–30 Hz. Continuous suction facilitated fragment evacuation and maintained low intrarenal pressure. Complete stone clearance was confirmed endoscopically and by intraoperative ultrasound. A 6-Fr antegrade ureteral stent was inserted, and the procedure was completed without nephrostomy tube placement.

The operative time was 90 min, and the estimated volume of blood lost was 10 mL. The postoperative course was uneventful. A postoperative renal and bladder ultrasound confirmed complete stone clearance and that the stent was appropriately positioned. The patient was discharged on postoperative day three. The ureteral stent was removed four weeks later without complications. A three-month postoperative ultrasound showed no residual stones or hydronephrosis. At follow-up, the contralateral 3.5 cm staghorn calculus was scheduled for staged fluoroscopy-free mini-PCNL using a similar CT–ultrasound fusion-guided approach.

## 3 Discussion

In this case, we utilized CT–ultrasound fusion imaging during a mini-PCNL to overcome limitations associated with standard and ultrasound-guided PCNL techniques. This involved integrating detailed preoperative anatomical information derived from CT with real-time ultrasound images.^13^^,^^14^ The resultant overlay functioned as a “virtual roadmap” and enabled us to confidently and accurately puncture the calyx without fluoroscopy.^15^ This approach was particularly valuable for targeting the upper-pole calyx, which might have been challenging using ultrasound alone.

Recent studies conducted with adult populations have demonstrated that CT–ultrasound fusion-guided PCNL can reduce the operative time and complications while improving access accuracy.^11^^,^^12^ Our case extends these findings to the pediatric setting, illustrating its feasibility and safety in a child with complex, recurrent cystine stones.

Cystine stones are typically hard and resistant to conventional lithotripsy.^2^^,^^16^ Hence, it is critical to select an effective and efficient stone fragmentation method when treating cystinuria. In this case, the use of thulium fiber laser lithotripsy combined with a suction access sheath facilitated effective stone fragmentation, continuous debris evacuation, and low intrarenal pressure. The latter is an important consideration for infection prevention in pediatric patients.^17^

Despite these advantages, CT–ultrasound fusion imaging has some limitations, including additional cost, the need for preoperative CT imaging, and a learning curve for image co-registration. Therefore, this technique should be reserved for selected cases in which ultrasound-only guidance is anticipated to be challenging rather than as a routine replacement.

## 4 Conclusion

Our findings indicate that fluoroscopy-free mini-PCNL with CT–ultrasound fusion imaging guidance is a safe and feasible technique for selected pediatric patients with complex renal stone disease. By enabling precise renal access without intraoperative radiation, this approach abides by the ALARA principle and is a valuable adjunct to ultrasound-guided PCNL in pediatric endourology.

## CRediT authorship contribution statement

**Surawach Piyawannarat:** Writing – original draft, Project administration, Formal analysis, Data curation, Conceptualization. **Kun Sirisopana:** Methodology, Investigation. **Yada Phengsalae:** Methodology, Investigation. **Premsant Sangkum:** Validation, Supervision. **Wisoot Kongchareonsombat:** Validation, Supervision. **Chinnakhet Ketsuwan:** Writing – review & editing, Supervision.

## Ethical approval

This case report was conducted in accordance with the ethical standards of the institution and the 1964 Helsinki Declaration and its later amendments or comparable ethical standards (ethical approval number: COA No. MURA2025/717). Informed consent was obtained from the patient.

## Funding

This research received no specific grant from any funding agency in the public, commercial, or not-for-profit sectors.

## Declaration of competing interest

The authors declare that they have no conflicts of interest.
